# General methods for measuring and comparing medical interventions in childbirth: a framework

**DOI:** 10.1186/s12884-020-02945-5

**Published:** 2020-05-07

**Authors:** Alessandro Svelato, Antonio Ragusa, Piero Manfredi

**Affiliations:** 1 Department of Obstetrics and Gynecology, San Giovanni Calibita Fatebenefratelli Hospital, Isola Tiberina, Rome, Italy; 2 Department of Economics and Management, University of Pisa, Pisa, Italy

**Keywords:** Labour, Intrapartum management, Cesarean section, Augmentation, Amniotomy, Oxytocin, Nulliparity, Multiparity, Intervention measures, Progression proportions, Robson classification

## Abstract

**Background:**

The continue increase of interventions during labour in low risk population is a controversial issue of the current obstetric literature, given the lack of evidence demonstrating the benefits of unnecessary interventions for women or infants’ health. This makes it important to have approaches to assess the burden of all medical interventions performed.

**Methods:**

Exploiting the nature of childbirth intervention as a staged process, we proposed graphic representations allowing to generate alternative formulas for the simplest measures of the intervention intensity namely, the overall and type-specific treatment ratios. We applied the approach to quantify the change in interventions following a protocol termed Comprehensive Management (CM), using data from Robson classification, collected in a prospective longitudinal cohort study carried out at the Obstetric Unit of the Cà Granda Niguarda Hospital in Milan, Italy.

**Results:**

Following CM a substantial reduction was observed in the Overall Treatment Ratio, as well as in the ratios for augmentation (amniotomy and synthetic oxytocin use) and for caesarean section ratio, without any increase in neonatal and maternal adverse outcomes. The key component of this reduction was the dramatic decline in the proportion of women progressing to augmentation, which resulted not only the most practiced intervention, but also the main door towards further treatments.

**Conclusions:**

The proposed framework, once combined with Robson Classification, provides useful tools to make medical interventions performed during childbirth quantitatively measurable and comparable. The framework allowed to identifying the key components of interventions reduction following CM. In its turn, CM proved useful to reduce the number of medical interventions carried out during childbirth, without worsening neonatal and maternal outcomes.

## Background

The continuous increase of obstetrical interventions during labour in low risk population is becoming one of the most discussed topics of the current obstetric literature [1–5]. The main interventions performed during childbirth include caesarean section, operative vaginal delivery and the use of (either or both) synthetic oxytocin and amniotomy. In-depth analysis of both UK and Italian data, [6, 7] highlighted a considerable variability in the ratio of caesarean sections among different geographic areas and even among different hospitals of the same area. This variability shows that, alongside the characteristics of the population under study, the existing heterogeneity in clinical practices can play a major role on the number of obstetric interventions during labour. When medically indicated, interventions can prevent maternal and perinatal mortality and morbidity. There is no evidence, however, demonstrating the benefits of interventions during labour for women or infants’ health when unnecessary [2, 8–10]. Rather, caesarean section is associated with short- and long-term risk for mother and child, which can extend beyond the current delivery and affect future pregnancies [2, 11–13]. The determinants of the increase in medical interventions are not fully understood, but emerge as a multifactorial combination of causes involving health systems, health care providers, women, societies, and even fashion and media, [14–16] to the extent that the labour ward environment and the intervening processes have been described as a complex system [17]. Given this complicate picture, it is critically important to assess the burden of all medical interventions, not just caesarean section, performed during childbirth.

The objective of the present work is to develop a framework integrated with Robson classification to quantify and to compare the intervention intensity during labour.

## Methods

Data were collected in a prospective longitudinal cohort study aimed to evaluate the effects of a novel labour–ward management protocol that we termed Comprehensive Management (CM) [18], carried out at the Obstetric Unit of the Cà Granda Niguarda Hospital in Milan, Italy, from 1 January 2012 to 31 December 2013. The study aimed at comparing intervention intensity before (henceforth denoted as the Before Comprehensive Management (BCM) group) and after (henceforth denoted as the After Comprehensive Management (ACM) group) the introduction of CM. The study was made up of 3 phases (Table 1).

**Table 1:** The three phases of the study

PHASES OF THE STUDY					
PHASE N°	START	END	GROUP	DESCRIPTION OF ACTIONS	N° PATIENTS
1	01/2012	06/2012	**BCM**	Data Collection and analysis.	**637**
2	07/2012	12/2012	**–**	Training of obstetric staff in view of introduction of CM; no data collection	**–**
3	01/2013	12/2013	**ACM**	Data Collection and analysis	**1375**
**TOT patients**					**2012**

The collected labour ward data were analysed by using Robson classification (Table 2).

**Table 2:** Summary of the collected labour ward data using the Robson’s classification

BCM group	N° women	CS	Relative size of groups (%)	CS rate in each group (%)	Contriution made by each group to the overall CS rate (%)	Percent of all cesarean sections (%)
GROUP I	245	20	26,9	8,2	2,2	7
GROUPIIa	107	36	11,7	33,6	3,9	12,6
GROUP IIb	43	43	4,7	100	4,7	15,1
GROUP III	232	3	25,4	1,3	0,3	1
GROUP IVa	53	6	5,8	11,3	0,7	2,1
GROUP IVb	1	11	1,2	100	1,2	3,9
GROUP V	99	79	10,9	79,8	8,7	28
GROUP VI	34	34	3,7	100	3,7	12
GROUP VII	10	7	1,1	70	0,8	2
GROUP VIII	18	15	2,0	83,3	1,6	5
GROUP IX	2	2	0,2	100	0,2	1
GROUP X	58	29	6,4	50	3,2	10
Total number of patients	912	285	100	31,3	31,3	100
ACM group	N° women	CS	Relative size of groups (%)	CS rate in each group (%)	Contriution made by each group to the overall CS rate (%)	Percent of all cesarean sections (%)
CLASSE I	580	35	30,4	6	1,8	7
CLASSE IIa	196	49	10,3	25	2,6	9,6
CLASSE IIb	41	41	2,2	100	2,2	8,1
CLASSE III	527	10	27,7	1,9	0,5	2
CLASSE IVa	72	4	3,8	5,6	0,2	0,8
CLASSE IVb	13	13	0,7	100	0,7	2,6
CLASSE V	196	165	10,3	84,2	8,7	32
CLASSE VI	44	44	2,3	100	2,3	9
CLASSE VII	33	29	1,7	87,9	1,5	6
CLASSE VIII	65	54	3,4	83,1	2,8	11
CLASSE IX	11	11	0,6	100	0,6	2
CLASSE X	127	54	6,7	42,5	2,8	11
Total number of patients	1905	509	100	26,7	26,7	100

We decided to focus on nulliparous or multiparous women, at term, with single cephalic baby in either spontaneous or induced labor (Robson classes I, IIa, IIIa e IV) [19]. This choice was motivated by the fact that these groups include women with a larger probability to give rise to a vaginal birth on condition that appropriate assistance during labour is provided.

Labour management characteristics in BCM and ACM are described elsewhere [18] and summarized in Table 3.

**Table 3:** Main differences in labour management between BCM and ACM [18]

Before Comprehensive Management (BCM)	After Comprehensive Management (ACM)
No regular labour monitoring, documentation of events, audit and feedback	Regular labour monitoring, documentation of events, audit and feedback [20]
No use of intrapartum ultrasound	Use of intrapartum ultrasound
Routine supine posture during labour, with consequent limited maternal movement	Mobility in labour and birth posture of women choice (women were encouraged to use preferred postures, to freely walk during labour and to give birth in the more comfortable position) [20]
Interpretation of cardio-tocography was left to personal interpretation of midwifes and physicians	Introduction of a formal classification of abnormal cardio-tocography in labour [21]
The women’s psychological and nutritional wellbeing were not taken into account	Continuity of care; respectful labour and childbirth care; Emotional support from a person of choice; oral fluid and food intake [20]
Standard use of partograph as a diagnostic tool for dystocia, systematic use of Fridman’s Curves as normality, following “one-centimeter per hour rule”	Partograph conceived as a screening tool without use of standard normality curves. As duration of the different stages of labour has not been established and can vary widely from one woman to another, we decided to be respectful of individual woman time.
Epidural analgesia given only upon woman’s request	Use of epidural analgesia, not only upon woman’s request, but also upon medical indication. Use of non-pharmaceutical methods of pain relief

### Obstetric interventions

We focused on four interventions: amniotomy, synthetic oxytocin use, ventouse and caesarean section. In the BCM group, labour augmentation was administered under a *strictly sequential protocol*, with amniotomy use always preceding that of synthetic oxytocin. On the other hand, in the ACM group the augmentation protocol was not sequential, so that it was allowed to initiate oxytocin perfusion before amniotomy. Therefore, for making the comparison between the BCM and ACM cohorts as homogeneous as possible, we decided to consider amniotomy and synthetic oxytocin use as a single intervention, termed *augmentation*, and focused on the three main interventions augmentation (A), ventouse (V), and caesarean section (C). Finally, the Apgar score and the pH on the umbilical artery, along with the main adverse neonatal and maternal outcomes were recorded.

The adopted methods of induction included Foley catheter, Dinoprostone and Oxyitocin, selected according to Bishop score, without differences in the induction protocol between the BCM and ACM groups. With the term augmentation we referred to the administration of oxytocin or amniotomy (or both) after the diagnosis of labour onset (18), aimed to speed up labour itself, regardless women’s labour was induced or spontaneous. Clinical diagnosis of active labor was made when the uterine cervix was effaced, dilated at least 4 cm and concomitant efficient contractile activity.

### Measuring interventions during labour: a framework

Here we describe a framework for measuring and comparing interventions during labour. The reference background is essentially demographic. Technical notes and references are reported in the Additional file 1.

**Fig. 1:**
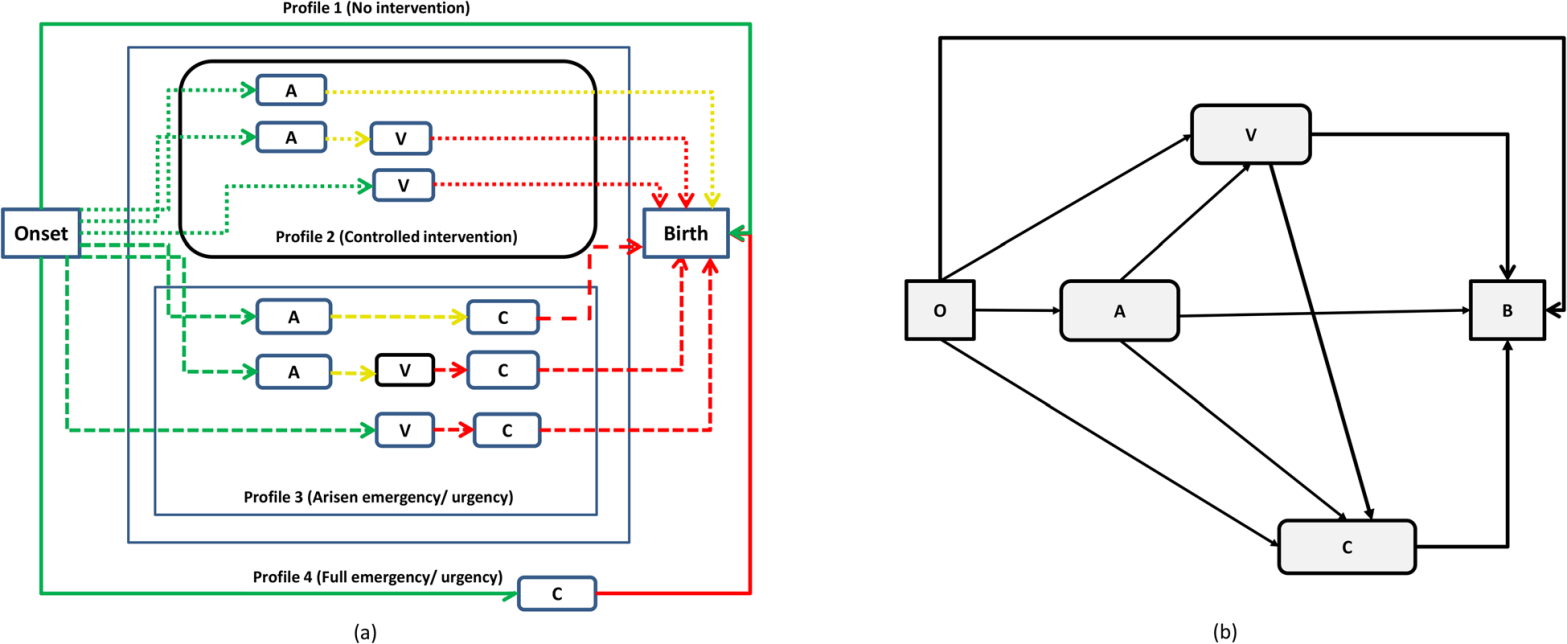
**a** Flowchart describing the different paths during labour for a situation where three main sequential interventions (A, V, C) are considered. Homogeneous paths are grouped into profiles. Legend: green continuous line = the no intervention profile; dotted lines = the controlled intervention profile; dashed line = arisen emergency/urgency; red continuous line = full emergency/urgency with direct transition to C. **b** Flowchart reporting the corresponding *compartmental* (or Markovian) representation. (A: Augmentation; V: Ventouse; C: Caesarean section; O: Onset; B: Birth)

During labour, taken as the process initiating with onset (O) of contractile activity and ending with a birth (B), a woman can receive different combinations of medical interventions. We assume that interventions are administered one at time under a sequential protocol, meaning that for women who received different interventions less invasive treatments always preceded more invasive ones, except for urgency and emergency. Under three sequential interventions, as considered here (i.e., A, V, and C), a hypothetical woman can follow any among 2^3^ = 8 (2^n^ if there are at most *n* sequential interventions) different, mutually exclusive and exhaustive, possible *intervention paths* (Fig. 1a), representing a combination of different interventions. All intervention paths in Fig. 1a can be represented by a sequences (*x*, *y*, *z*) of three binary variables, each one taking value 0 if the woman did not experience the intervention considered and 1 if she did, where the sum (*x* + *y* + *z*) represents the number of interventions experienced in total by a woman following path (*x*, *y*, *z*). For instance, a woman who experienced augmentation and ventouse but not caesarean section would be represented as (*x* = 1, *y* = 1, *z* = 0), with 1 + 1 + 0 = 2 interventions in total.

Another useful representation is the *compartmental* one (Fig. 1b), focusing on the fact that a woman can pass through different intervention *compartments or states, and often used to* represent *Markovian* systems. Markovian systems are characterised by the property that the probability to move from the current state to a different one only depends on being in the current state and not on the past history. The idea, implicit in Fig. 1b, is that for example the probability that a woman moves from state V to state C, only depends on “being in V” i.e., the possible path yielding to V are not relevant for the staff decision to move to caesarean section. This is the case when e.g., urgency forces the medical staff to take on sequential decisions based only on the current information about the maternal and foetal state. The Markov representation reflects a precise hypothesis and therefore is more restrictive than the descriptive representation of Fig. 1a. Consequently, in actual situations it is not necessarily true but should instead be tested.

Consider a group of *N* women who received *E* interventions in total. The simplest measure of the intensity of medical intervention is the *overall treatment ratio* (OTR): *H* = *E*/*N*, representing the average number of treatments per woman (including untreated women). It ranges between 0 (when no women are treated) and n (when all women receive all treatments). By dividing the OTR by n we obtain the iatrogenic labor index (ILI, 0 ≤ ILI ≤ 1) [18], a normalized measure relating total interventions to the maximal number of interventions hypothetically administrable to all women (n•N). Both OTR and ILI have this meaning: a “small” value means that a few intrapartum interventions occurred. Though OTR and ILI can be straightforwardly used for comparisons i.e., if in site 2 (or time 2) the OTR took a value *H*_2_ larger than the value *H*_1_ observed in site 1 (time 1), this means that women in site 2 received “more treatments” on average than in site 1, they values say little on the factors that determined the *observed difference H*_2_ − *H*_1_. Table 4 reports (for the case of three interventions A,V,C), some alternative representations for *H* that are more informative for comparison purposes than the mere inspection of the values of H, because they highlight different facets of intervention and the related components: (i) form 1, highlighting the proportion of women who received some treatment, (ii) form 2, decomposing the OTR as the sum of the *type-specific treatment ratios* (STR) i.e., computed for each type of intervention A,V,C, (iii) form 3, representing *H* as the average of the distribution of the number of treatments received, (iv) form 4, representing *H* as the average of the distribution of the number of treatments along the different intervention paths of Fig. 1a, and finally (v) form 5, focusing on the Markov representation of Fig. 1b, and expressing *H* in terms of the *progression proportions* (PP), which specify the proportions of women progressing to a further treatment from current treatment.

**Table 4:** The alternative forms for the OTR (overall treatment ratio) in the case of three interventions (A = augmentation, V = ventouse, C = cesarean section)

**Form 1**	***Treatment ratio for treated women.*** Letting *N*_0_ denote the number of women who did not receive any treatments, *S* = (*N* − *N*_0_)/*N* is the proportion of women (0 ≤ S ≤ 1) who received some treatment, or *treated proportion*. The OTR can then be defined as: $H=\frac{E}{N}=\frac{N-{N}_0}{N}\bullet \frac{E}{N-{N}_0}=S\bullet {H}_T$ i.e., as the product of S times the average number of treatments (*H*_*T*_) among treated women.
**Form 2**	***OTR as the sum of type-specific treatment ratios*.** A shortcoming of OTR is that it does not distinguish between different types of interventions i.e., the same OTR can be obtained with either a large number of augmentations or a large number of cesareans sections, possibly with different health outcomes. Letting *E*_*A*_, *E*_*V*_, *E*_*C*_ (*E*_*A*_ + *E*_*V*_ + *E*_*C*_ = *E*) denote the number of interventions of type A,V,C respectively, it holds: $H=\frac{E_A+{E}_V+{E}_C}{N}$= *H*_*A*_ + *H*_*V*_ + *H*_*C*_ where *H*_*A*_, *H*_*V*_, *H*_*C*_ are the type-*specific treatment ratios* (STR) i.e., the proportions of women treated by augmentation, ventouse, and cesarean section, respectively.
**Form 3**	***OTR as the average of the distribution of the number of treatments.*** Let *N*_0_, *N*_1_, *N*_2_, *N*_3_ (*N*_0_ + *N*_1_ + *N*_2_ + *N*_3_ = *N*) denote the number of women who received i = 0,1,2,3 treatments respectively, and *f*_0_ = *N*_0_/*N*, *f*_1_ = *N*_1_/*N*, *etc* the corresponding proportions. Then: $H=\frac{E}{N}=\frac{1\bullet {N}_1+2\bullet {N}_2+3\bullet {N}_3}{N}$= 1 ∙ *f*_1_ + 2 ∙ *f*_2_ + 3 ∙ *f*_3_ In this form H is the average of the statistical distribution of the number of treatments women received.
**Form 4**	***OTR as the average of the distribution of the number of treatments along the different intervention paths in*** Fig. 1a. Let *N*(*x*, *y*, *z*) and *f*(*x*, *y*, *z*) = *N*(*x*, *y*, *z*)/*N*, respectively denote the number and the proportion of women who followed path (x,y,z), therefore receiving (x + y + z) treatments. Then H is simply the average of the number of treatments along each path weighted by the proportion of women who followed that path: $H=\sum \limits_{x=0}^1\sum \limits_{y=0}^1\sum \limits_{z=0}^1\left(x+y+z\right)\bullet f\left(x,y,z\right)$ By aggregating paths with the same number of treatments Form 4 collapses into Form 3.
**Form 5**	***OTR as a function of the progression proportions*.** Under the assumptions of Fig. 1b, the STRs can be expressed in terms of the *progression proportions* (PP), which specify the proportions of women progressing to a further treatment from current treatment. For example the STR for ventouse *H*_*V*_ is given by the sum of the PP *p*_*OV*_ of women who entered labour (O) and progressed directly to ventouse (V) (the direct path OV in Fig. 1b), therefore receiving exactly one treatment, plus the proportion of women who experienced augmentation before ventouse (receiving exactly two treatments), which can be factored as the product *p*_*OA*_*p*_*AV*_ of the PP to augmentation *p*_*OA*_ times the PP *p*_*AV*_ from augmentation to ventouse (the two-steps path from O to A and from A to V). By this reasoning we can write STRs *H*_*A*_, *H*_*V*_, *H*_*C*_ as: *H*_*A*_ = *p*_*OA*_ *H*_*V*_ = *p*_*OV*_ + *p*_*OA*_*p*_*AV*_ *H*_*C*_ = *p*_*OC*_ + *p*_*OA*_*p*_*AC*_ + *p*_*OV*_*p*_*VC*_ + *p*_*OA*_*p*_*AV*_*p*_*VC*_ Recalling form 2, the OTR can then be represented as: *H* = *p*_*OA*_ + *p*_*OV*_ + *p*_*OA*_*p*_*AV*_ + *p*_*OC*_ + *p*_*OA*_*p*_*AC*_ + *p*_*OV*_*p*_*VC*_ + *p*_*OA*_*p*_*AV*_*p*_*VC*_

Using alternative representations, as forms 1,2,..,5, depending on different underlying components, or “factors”, allows to quantify which components, contributed more to an observed difference (*H*_2_ − *H*_1_) (e.g., over two different time periods or two different settings) by focusing on different facets of intervention with increasing level of detail. We illustrate this considering Forms 1 and 5 for brevity.

Sub Form 1 the CTR is the product of the treated proportion (**S**) and the treatment ratio among treated women (*H*_*T*_) and we would like to establish whether the difference *H*_2_ − *H*_1_ was primarily due to the change in *S* or to the change in *H*_*T*_. In this simple case the *relative difference* (*H*_2_ − *H*_1_)/*H*_1_ can be decomposed into the sum of the relative change in *S* plus the relative change in *H*_*T*_ plus the product of the two changes, reflecting the interaction between the two factors. This allows to exhaustively characterize the role of the two factors.

Sub Form 5 we would like to assess which progression proportions contributed more to the difference *H*_2_ − *H*_1_. In this case the simplest general approach consists in the *stepwise* replacement of each PP in the computation of *H*_1_ with the analogous term in *H*_2_. Briefly, as a first step, one replaces the first PP of *H*_1_ (say, ${p}_{OA}^1$), by the first element of *H*_2_ (${p}_{OA}^2$), obtaining a new quantity ${H}_1^{\ast }$. The difference ${H}_1^{\ast }-{H}_1$ represents the contribution to the difference *H*_2_ − *H*_1_ due to the change of the first PP only. Then one continues by replacing the second element in *H*_1_ by the corresponding element in *H*_2_, etc., until all elements have been replaced, thereby eventually transforming *H*_1_ into *H*_2_. As a result, *H*_2_ − *H*_1_ is decomposed as a sum of “partial” differences each one representing the effect of each single replacement, and one can look which partial differences contributed more to the overall difference *H*_2_ − *H*_1_.

### Robson classification and the present framework

The use of previous representations for identifying the components of the difference in the OTR observed e.g., in different settings or at different time points, will be especially effective when the groups of women under comparison are sufficiently homogeneous i.e., factors potentially promoting differences are kept under control. As in the obstetrics sciences there is a widely acknowledged tool, namely Robson classification, allowing to split women into homogeneous classes with respect to inner heterogeneity factors, the optimal situation would be to use the present approach, whenever available data allow to do so, for making comparisons between single Robson classes. Nonetheless, the approach can also be used when different Robson classes are aggregated together, by controlling for the differences in the proportions of women in the various Robson classes, by means of ordinary standardization procedures. From this standpoint, the present framework can be considered as complementary to the concept of Robson classification; the latter represents the natural environment within which to cast the baseline methodology proposed here.

## Results

### Background facts

We recruited 2012 patients in total, of which 637 from the BCM group and 1375 from the ACM group (Table 1). Table 5 reports background information on women from the two study groups, which were found to be comparable for all the variables under consideration.

**Table 5:** Background information of the two groups (BCM vs ACM) of women taking part in the study (SD: standard deviation; NS: not significant)

***Basic and demographic data***	***BCM Group***	***ACM Group***	***Difference***
Number of women	637	1375	
Nulliparous (%)	352 (55.3)	776 (56.4)	NS
Multiparous (%)	285 (44.7)	599 (43.6)	NS
Average age, in years [SD]	31,7 [5,4]	31,9 [5,7]	NS
Average gestational age, in weeks [SD]	39,9 [1,1]	40,1 [1,1]	NS
Range og gestational age, in weeks	37–42 ws	37–42 ws	NS
Previous abortions (%)	172 (27)	369 (27)	NS
Previous stillbirth	0	0	NS
Previous preterm birth	0	0	NS
Average fetal weight, in grammes at birth [SD]	3362 [416]	3337 [425]	NS
Ethnicity			
Italian (%)	465 (73)	930 (68)	NS
Other (%)	172 (27)	445 (32)	NS
Epidural use (%)	146 (23)	297 (22)	NS

Perinatal and maternal adverse events were comparable in either of the two groups of the study (Table 6).

**Table 6:** Main adverse neonatal and maternal outcomes

***Perinatal and maternal adverse event***	***BCM Group***	***ACM Group***	***Difference***
Episiotomy (%)	230 (36.1)	409 (29.7)	NS
Third and fourth degree lacerations (%)	3 (0.5)	6 (0.4)	NS
Major postpartum hemorrhage (≥1500 ml) (%)	12 (1.9)	30 (2.2)	NS
Number of blood transfusions (%)	0 (0)	2 (0.1)	NS
Maternal mortality	0	0	NS
Maternal intensive care unit admission	0	0	NS
Newborns with an Apgar score at 5 min of 7 or less and/or pH of the umbilical artery of 7.00 (composite measure) (%)	6 (0.9)	7 (0.5)	NS
HIE	0	0	NS
Perinatal death	0	0	NS

### Comparing interventions in the BCM and ACM cohorts

Given the mainly methodological aim of the present article and for a sake of simplicity, in what follows we present our main results in a simplified form, by commenting only the aggregated differences betweeen the overall BCM and the ACM cohorts i.e., by taking together the women belonging to the four Robson classes considered. Nonetheless, detailed results on each single Robson class are reported in the Additional file 1.

The STR for augmentation (*H*_*A*_) decreased by 31.6%, passing from 33.8% (215/637 patients) in the BCM group to = 23.1% (317/1375) in the ACM group (*p*-value< 0.0001). The STR for C (*H*_*C*_) also decreased by 30%, from 10.2% (65/637 patients) in the BCM group to 7.1% (98/1375 patients) in the ACM cohort (p-value< 0.02). Looking more specifically at the four Robson classes considered, we noted a decrease in the number of caesarean section in classes I, IIa and IVa, and an increase in class III. The STR for V (*H*_*V*_) increased by 10.2% passing from 4.2% (27/637 patients) in the BCM group to 4,8% in the ACM group (66/1375 patients) (difference not significant).

As regards the intervention paths of Fig. 1a, Table 7 shows that out of the four paths more contributing to the intervention burden in the BCM group, large declines were allowed by ACM on the paths (1,0,0) (“augmentation only”), (1,0,1) (“augmentation plus caesarean section”), and (1,1,0), (“augmentation plus ventouse”) (all changes significant) while the frequency of direct transitions to caesarean section, path (0,0,1), declined only marginally.

**Table 7:** Effects of Comprehensive Management as reflected by the changes in the percentage of women following the different intervention paths (x,y,z) in Fig. 1a

Path (x,y,z)	Number of interventions (x + y + z)	BCM		ACM		
		Number of women N(x,y,z)	%	Number of women N(x,y,z)	%	Relative difference (%)
(0,0,0)	0	387	60,8	969	70,5	16,0
(1,0,0)	1	160	25,1	245	17,8	−29,1
(0,1,0)	1	9	1,4	36	2,6	85,3
(0,0,1)	1	25	3,9	51	3,7	−5,5
(1,1,0)	2	16	2,5	27	2,0	−21,8
(1,0,1)	2	38	6,0	44	3,2	−46,4
(0,1,1)	2	1	0,2	2	0,1	−7,3
(1,1,1)	3	1	0,2	1	0,1	− 53,7
**Total**		637	100,0	1375	100,0	

Differences in the distributions of women across the different paths between the BCM and ACM cohorts were tested by the ordinary chi-square test for the difference between multinomial distributions, under the standard caveats (e.g., that each class contains a minimal number of data etc). The observed difference between the BCM and ACM pathways distributions reported in Table 7 resulted highly significant.

### Identifying main components of observed differences

We now report results of our approach aimed to identify the factors contributing to the observed difference in the OTR. Here, we only focus on the aggregated cohors, but detailed results for each Robson class are reported in the Additional file 1.

We focus, for sake of brevity, on Forms 1,5. Since now on our focus will be on the procedure rather than on statistical significance. Women in the two groups experienced 307 (BCM) and 481 (ACM) medical treatments respectively, causing the OTR to decline from *H*_1_= 48.1 interventions on average per 100 women (BCM) to *H*_2_ =34.9 (ACM), with a percentage decline of 27.4%. Based on Form 1, the major part of this decline resulted from the proportion (S) of treated women, which fell by about 25%, from 39.2% (BCM) to 29.5% (ACM), while the treatment intensity (H_T_) among treated women contributed marginally, by declining from 1.23 to 1.18 treatments per women (− 3.5%), with a negligible contribution from the interaction term.

The analysis, by Form 5, of which PPs mostly contributed to the decline of the OTR, is summarised in Table 8, whose upper block reports the steps of the replacement algorithm. The first column lists the various PPs involved. Column two reports the values of the PPs observed in the BCM group which, once combined by Form 5 yield the corresponding value of the OTR (H), reported in the “H” row. Pairwise, the last column reports the PPs values in the ACM group, supplemented by the relative rates of change compared to the BCM group. In column three, the replacement algorithm starts, by replacing (shadowed cell) the value of the first PP (*p*_*OA*_) of BCM group (*p*_*OA*_= 0,338) by the corresponding value in the ACM group (0,231). In column four, the algorithm continues by replacing also the values of the second PP (*p*_*OV*_) of BCM group (0,016) by the corresponding ACM value (0,028), etc. The algorithm ends when all the values of the PPs in the BCM cohort have been replaced. The replacement order adopted in Table 8 is the natural one by which, given that a woman at any stage of labour can move to different treatments, we replace first those PPs leading to less invasive treatments.

**Table 8:** Illustration of the stepwise replacement procedure and contributions of progression proportions to the overall decline (between BCM and ACM groups) of: (i) the crude treatment ratio *H*, (ii) the treatment ratio for caesarean section, *H*_*C*_, and (iii) the treatment ratio for operative delivery, *H*_*VC*_. The values of *H*, *H*_*C*_, *H*_*VC*_ are based on Form 5 in Table 4

	Cohort 1 (BCM)	Replace *p*_*OA*_	& replace *p*_*OV*_	& replace *p*_*OC*_	& replace *p*_*AV*_	& replace *p*_*AC*_	& replace *p*_*VC*_	Cohort 2 (ACM)
*p*_*OA*_	0,338	0,231	0,231	0,231	0,231	0,231	0,231	0,231 (−31.7%)
*p*_*OV*_	0,016	0,016	0,028	0,028	0,028	0,028	0,028	0,028 (+ 76%)
*p*_*OC*_	0,039	0,039	0,039	0,037	0,037	0,037	0,037	0,037 (− 5.5%)
*p*_*AV*_	0,079	0,079	0,079	0,079	0,088	0,088	0,088	0,088 (+ 11.7%)
*p*_*AC*_	0,177	0,177	0,177	0,177	0,177	0,139	0,139	0,139 (−21.5%)
*p*_*VC*_	0,074	0,074	0,074	0,074	0,074	0,074	0,045	0,045 (−38.6%)
***H***	**0,482**	**0,347**	**0,360**	**0,358**	**0,360**	**0,351**	**0,350**	**0,350**
${H}_{i+1}^{\ast }-{H}_i^{\ast }$		−0,135	0,013	−0,002	0,002	−0,009	−0,001	
*Relative contributions to observed difference H*_2_ − *H*_1_		−100,00	9,50	−1,60	1,70	−6,48	−1,02	
*H*_*C*_	**0,102**	**0,083**	**0,083**	**0,081**	**0,081**	**0,073**	**0,071**	
${H}_{C,i+1}^{\ast }-{H}_{C,i}^{\ast }$		−0,020	0,001	−0,002	0,000	−0,009	−0,001	
*Relative contributions to observed difference*		−100,0	4,5	− 11,0	0,8	−44,8	−7,0	
*H*_*V*_ + *H*_*C*_	**0,144**	**0,116**	**0,129**	**0,127**	**0,129**	**0,121**	**0,119**	
${H}_{V,C,i+1}^{\ast }-{H}_{V,C,i}^{\ast }$		−0,028	0,013	−0,002	0,002	−0,009	−0,001	
*Relative contributions to observed difference*		−100,0	45,8	−7,7	8,2	−31,3	−4,9	

The “H” row reports the values of the OTR over the various steps i.e., for example, the value of 0,347 for column three, is the value of the OTR that results by replacing only the PP to augmentation in BI cohort by the corresponding ACM value. The row labelled as (${H}_{i+1}^{\ast }-{H}_i^{\ast }$) reports the differences resulting from subsequent replacements. Finally, the subsequent row (“Relative contributions to observed difference”) reports the relative contribution of each PP to the decline in OTR passing from the BCM to the ACM group, showing the disproportionate contribution of the change in the PP to augmentation *p*_*OA*_. Indeed, setting (for simplicity) to − 100 the contribution from *p*_*OA*_, the second factor most contributing to the decline, namely the proportion progressing from augmentation to caesarean section *p*_*AC*_, was 15 times lower (− 6,48). Notably, the *p*_*OV*_ term worked the other direction (+ 9,5).

The same methodology can be applied to decompose the decline in the STR for caesarean section, *H*_*C*_ (Table 8, “ *H*_*C*_ ” rows). This principally followed from the fall in the PP to augmentation *p*_*OA*_, and secondarily from the decline in the PP to caesarean section from augmentation (*p*_*AC*_), while was only slightly affected by the proportion progressing directly to caesarean section, *p*_*OC*_. Setting to (− 100) the contribution to the decline of *H*_*C*_ resulting from the decline in *p*_*OA*_, the contribution from *p*_*AC*_ was less than half of this (− 44,8), while the contribution from *p*_*OC*_ resulted ten times smaller (− 11,0), reflecting the fact that the proportion progressing directly to caesarean section only slightly changed between BCM and ACM groups.

Last, the change in the ratio *H*_*V*, *C*_ = *H*_*v*_ + *H*_*c*_ (Table 8, lower block), measuring the overall intensity of operative delivery (V + C), declined from 14.4 per 100 women (BCM) to 11.9 (ACM), with a relative decline of 17.4%, following from the partial compensation between the larger decline in *H*_*C*_ and the moderate increase in *H*_*V*_. Though the decline in the proportion progressing to augmentation remains the major source of the decline in *H*_*V*, *C*_ (− 100), the increase in *p*_*OV*_ had a substantial opposite effect (+ 46), larger than the contribution of the decline in *p*_*OC*_ (− 31).

## Discussion

Our results showed that comprehensive management was associated with a substantial reduction of the overall treatment ratio as well as the caesarean section ratio without adversely affecting the major neonatal and maternal outcomes. The decline in the overall caesarean section rate was principally due to the reduction of the proportion of treated women, with the maternal and foetal outcomes remained unaltered.

Women, physicians, midwives and policy makers demand methods to assess obstetric outcomes in the labour ward [22–24]. Assessing the quality of maternal care is made difficult by the intrinsic complexity of labour ward [17] that involve a high degree of specialization [25, 26].

By its very nature, the intervention during labour is a staged process where, after each treatment, the decision to progress to further treatments, depends on multifactorial factors in which predominant contributions are played by the woman’s feelings and her preferences, joint with the approach of the obstetric team and with external constraints. Consistently, in this work we have (i) proposed a graphic framework for representing the stages of intervention, (ii) proposed alternative representations of the simplest measures of the intensity of interventions, reflecting the various stages, (iii) applied the approach to quantify the most important components of the reduction of the interventions burden, observed in a large Northern-Italy hospital following a specifically adopted protocol (CM).

Summarizing deeper our results, the key component of the reduction in the intervention burden following CM, resulted the decrease of the proportion of women progressing to augmentation. Augmentation, was indeed not only the by far most practiced intervention, but also the main door towards further treatments. With all limitations of our field design, we believe that CM was fully successful in achieving its target of bringing a cultural and organizational change of the obstetric staff as a whole [27]. Key to this was giving full priority to the individual woman’s time needs during the first stage of labour, particularly in relation to the course of cervical dilatation [20]. Indeed, by giving women an adequate time [19], we were able to identify those subjects for which interventions were really indicated, therefore improving the degree of intervention appropriateness. In relation to the time issue, our previous results [19] are in line with those recent findings suggesting that the “one-centimetre per hour” rule may be misleading, and eventually harmful [20, 28–30]. Further, the longer time allowed to most women to reach the second phase of labour, changes the indications of operative delivery (data not reported). We believe that the small and not statistically significant increment in the use of ventouse found in our study, should be considered suggestive of the achieved cultural and organizational change, due to the acquired awareness, by the staff, that operative vaginal birth whenever possible, is preferable, in terms of health outcome, to cesarean section [2, 31].

Unfortunately, with the partial exception of cesarean sections, the obstetric literature lacks of reliable data with regards to the appropriateness of medical intervention during childbirth in the short and especially in the long term [8, 32, 33]. Brook stressed that “the concern regarding the increased complexity of medical treatment is, for some patients, the lack of necessary care and, in others, being subject to useless procedures” [34–36].

So far, much of the literature on labour intervention typically focused on differences in the ratios of augmentation, operative vaginal delivery and cesarean sections, and their relations with the characteristics of the population under study, clinical practice, organization of resources and local obstetric culture and practices [19, 27, 37]. We believe that a main strength of this work is that the presented framework, in association with Robson Classification, is a widely applicable tool, which aims to go beyond the elementary comparison between the previous ratios, by quantifying the ultimate components of the differences in the intensity of medical interventions during childbirth, in different labour ward settings. Robson classification and the present conceptual framework are complementary: the former is a robust classification tool, the second one represents a unified approach to intervention data using Robson Classification. These can allow a better understanding of interventions during childbirthd which can help to: (i) improve the evaluation of labour wards performances, (ii) collect better data, (iii) optimize intervention activities, (iv) prioritize overall management strategies aimed to avoid un-necessary interventions, and ultimately (v) improve the level of appropriateness of interventions in labour wards. On the other hand, we believe that CM as a cultural and organizational change of the obstetric team, aimed to remove “avoidable” interventions in labour, is an effective strategy replicable in different settings by low budget resources.

Besides data collection design, a limitation of the present work is that we did not investigate the level of mothers’ satisfaction in the two groups considered. This aspect is clearly central and has been the subject of a recently published study [38].

Moreover, in this manuscript we have considered only a few types of interventions. Future work should aim at investigating iatrogenic pathways by including further relevant medical interventions such as induction, epidural, episiotomy, etc.

The approach to the measurement of labour ward intervention proposed here, is based on three objective facts: 1) childbirth with a minimal level of intervention is the optimal solution for the maternal-neonatal health [39–42]; 2) labour ward is a complex setting requiring conceptual frameworks for collecting and analyzing data [17]; 3) even in the absence of optimal measures of appropriateness, the system of measures of medical intervention introduced here, are useful for the identification of the main components of medical intervention during childbirth, aiding to identify and properly adjust management targets. The proposed framework, combined with Robson classification, allows to make the interventions performed during childbirth, quantitatively measurable and comparable.

## Conclusions

The presented work can be considered a first step towards a rational approach to labour wards phenomena.

The proposed framework, optimally exploits Robson classification to make interventions performed during childbirth, quantitatively measurable and comparable.

Comprehensive management was associated with a substantial reduction of the overall treatment ratio as well as the caesarean section ratio without adversely affecting the major neonatal and maternal outcomes.

Further steps will aims at the statistical modelling of labor ward data, in order to predict the likely medical interventions which a woman may need during childbirth, based on individual characteristics, and at the mathematical modelling, to appropriately incorporate the time variable, central to account for labour ward dynamics and constraints.

## Supplementary information

**Additional file 1.**

## Data Availability

According to the regulations of the Ethics Committee and the Italian legislation, the clinical dataset generated and analyzed during the current study, cannot be made publicly available since that breeches local data protection laws. The data are however available from the corresponding author (AR) for inspection upon reasonable request.

## Abbreviations

CM: Comprehensive Management
BCM: Before Comprehensive Management
ACM: After Comprehensive Management
A: Augmentation
V: Ventouse
C: Caesarean section
OTR: Overall Treatment Ratio
ILI: Iatrogenic Labor Index
STR: Type-Specific Treatment Ratios
PP: Progression Proportions
