# Supplementary material for: General methods for measuring and comparing medical interventions in childbirth: a framework
Source: BMC Pregnancy Childbirth. 2020 May 7;20:279. doi: 10.1186/s12884-020-02945-5 (PMC7203888; doi:10.1186/s12884-020-02945-5)
Supplement: Supplementary file 1 — Additional file 1. [file 12884_2020_2945_MOESM1_ESM.docx]

**Appendix**

In this appendix, we report some further results (section A.1) and a number of technical details on the framework (section A.2), including references.

**A.1 Further results**

For the sake of completeness, we report detailed results for each single Robson class of some of the analyses that were presented in the main text using the data in aggregated form.

*Treatment ratios in each Robson class*

The overall ($H$) and specific (${H_{A},H}_{V},H_{C}$) treatment ratios were computed for each of the four Robson classes considered (1,2a,3,4a) and also for the aggregated classes 1 & 3 (spontaneous labour) and 2a & 4a (induced labour). Related results, including percentage change between the two cohorts, are reported in (Readers should interpret the Table with caution depending on the substantial differences in the number of women throughout the different classes). documents that the containment of acceleration (AMOX) was systematic in all Robson groups, in particular was dramatic in class 3 (-43%), and was substantial also in class 2a, where in the BCM cohort most women (3 out of four) received acceleration. As regards caesarean sections, a marked decline was obtained in classes 1,2a and 4, while an increase occurred in class 3, which by the way was the one characterised by the minimal resort to CS (one woman out of 100 in the BCM), thereby explaining the massive overall decline (-30%) also reported in the main text. As also noted in the main text, a small proportion of this gain was balanced by the specular -necessary - increase in the use of the ventouse.

|  |  | **Robson class** | | | | | | |
| --- | --- | --- | --- | --- | --- | --- | --- | --- |
|  |  | **1** | **2a** | **3** | **4a** | **1 & 3** | **2a & 4a** | **all** |
| **BCM** | $H_{A}$ | 0,29 | 0,75 | 0,16 | 0,47 | 0,23 | 0,66 | 0,34 |
| **ACM** | $H_{A}$ | 0,22 | 0,57 | 0,09 | 0,40 | 0,16 | 0,52 | 0,23 |
|  | % change | -23,8 | -24,7 | -43,2 | -14,6 | -30,0 | -20,9 | -31,8 |
| **BCM** | $H_{V}$ | 0,07 | 0,07 | 0,01 | 0,02 | 0,04 | 0,06 | 0,04 |
| **ACM** | $H_{V}$ | 0,08 | 0,08 | 0,01 | 0,00 | 0,05 | 0,06 | 0,05 |
|  | % change | 24,1 | 11,2 | -34,0 | -100,0 | 19,7 | 7,5 | 13,6 |
| **BCM** | $H_{C}$ | 0,08 | 0,33 | 0,01 | 0,11 | 0,05 | 0,26 | 0,10 |
| **ACM** | $H_{C}$ | 0,06 | 0,25 | 0,02 | 0,06 | 0,04 | 0,20 | 0,07 |
|  | % change | -26,1 | -24,3 | 46,7 | -50,9 | -15,7 | -23,7 | -29,9 |
| **BCM** | $H$ | 0,44 | 1,16 | 0,19 | 0,60 | 0,31 | 0,98 | 0,48 |
| **ACM** | $H$ | 0,36 | 0,90 | 0,12 | 0,46 | 0,25 | 0,78 | 0,35 |
|  | % change | -17,1 | -22,3 | -36,5 | -24,1 | -21,9 | -20,0 | -27,4 |
|  |  |  |  |  |  |  |  |  |

Table 1. Effects of Comprehensive Management as reflected by the % changes in the Treatment ratios between the BCM and ACM cohorts, for each of the four Robson classes considered (1,2a,3,4a), and for the aggregated classes (1,3) (“spontaneous labour”) and (2a,4a) (“induced labour”) considered together. For reference purpose also the figures (reported in the main text) for the four classes aggregated together are added (column “all”). We report here for ease the number of women in each Robson class (as detailed in Table 2 of the main text). BCM cohort: class 1: 245; class 2a: 109; class 3: 232; class 4a: 53. ACM cohort: class 1: 580; class 2a: 196; class 3: 527; class 4a: 72.

*Changes in the different treaments paths*

Still for completeness we report (in analogy with Table 5 of the main text) the effects of Comprehensive Management on the different intervention paths separately for each Robson class () as well as for the aggregated classes 1 & 3 (spontaneous labour) and 2a & 4a (induced labour) (). Obviously, some paths are very rare making the related comparison trivial. For example, allows to appreciate that much of the increase in the use of ventouse, that Table 7 of the main text showed to occur along path (0,1,0), was mostly concentrated in Robson class one.

| ***Robson class*** | ***Intervention path (x,y,z)*** | ***Number of interventions (x+y+z)*** | ***BCM*** | | ***ACM*** | | ***% change*** |
| --- | --- | --- | --- | --- | --- | --- | --- |
|  |  |  | *Number of women N(x,y,z)* | *% of women f(x,y,z)* | *Number of women N(x,y,z)* | *% of women f(x,y,z)* |  |
| *1* | *(0,0,0)* | *0* | *157* | *64,08* | *398* | *68,6* | *7,08* |
| *1* | *(0,0,1)* | *1* | *12* | *4,90* | *22* | *3,8* | *-22,56* |
| *1* | *(0,1,0)* | *1* | *5* | *2,04* | *31* | *5,3* | *161,90* |
| *1* | *(0,1,1)* | *2* | *0* | *0,00* | *1* | *0,2* | *-* |
| *1* | *(1,0,0)* | *1* | *53* | *21,63* | *101* | *17,4* | *-19,50* |
| *1* | *(1,0,1)* | *2* | *7* | *2,86* | *12* | *2,1* | *-27,59* |
| *1* | *(1,1,0)* | *2* | *10* | *4,08* | *15* | *2,6* | *-36,64* |
| *1* | *(1,1,1)* | *3* | *1* | *0,41* | *0* | *0,0* | *-100,00* |
| *2a* | *(0,0,0)* | *0* | *17* | *15,60* | *64* | *32,7* | *109,36* |
| *2a* | *(0,0,1)* | *1* | *8* | *7,34* | *16* | *8,2* | *11,22* |
| *2a* | *(0,1,0)* | *1* | *1* | *0,92* | *4* | *2,0* | *122,45* |
| *2a* | *(0,1,1)* | *2* | *1* | *0,92* | *1* | *0,5* | *-44,39* |
| *2a* | *(1,0,0)* | *1* | *49* | *44,95* | *69* | *35,2* | *-21,69* |
| *2a* | *(1,0,1)* | *2* | *27* | *24,77* | *31* | *15,8* | *-36,15* |
| *2a* | *(1,1,0)* | *2* | *6* | *5,50* | *10* | *5,1* | *-7,31* |
| *2a* | *(1,1,1)* | *3* | *0* | *0,00* | *1* | *0,5* | *-* |
| *3* | *(0,0,0)* | *0* | *190* | *81,90* | *467* | *88,6* | *8,20* |
| *3* | *(0,0,1)* | *1* | *2* | *0,86* | *10* | *1,9* | *120,11* |
| *3* | *(0,1,0)* | *1* | *2* | *0,86* | *1* | *0,2* | *-77,99* |
| *3* | *(0,1,1)* | *2* | *0* | *0,00* | *0* | *0,0* | *-* |
| *3* | *(1,0,0)* | *1* | *37* | *15,95* | *47* | *8,9* | *-44,08* |
| *3* | *(1,0,1)* | *2* | *1* | *0,43* | *0* | *0,0* | *-100,00* |
| *3* | *(1,1,0)* | *2* | *0* | *0,00* | *2* | *0,4* | *-* |
| *3* | *(1,1,1)* | *3* | *0* | *0,00* | *0* | *0,0* | *-* |
| *4a* | *(0,0,0)* | *0* | *24* | *45,28* | *40* | *55,6* | *22,69* |
| *4a* | *(0,0,1)* | *1* | *3* | *5,66* | *3* | *4,2* | *-26,39* |
| *4a* | *(0,1,0)* | *1* | *1* | *1,89* | *0* | *0,0* | *-100,00* |
| *4a* | *(0,1,1)* | *2* | *0* | *0,00* | *0* | *0,0* | *-* |
| *4a* | *(1,0,0)* | *1* | *22* | *41,51* | *28* | *38,9* | *-6,31* |
| *4a* | *(1,0,1)* | *2* | *3* | *5,66* | *1* | *1,4* | *-75,46* |
| *4a* | *(1,1,0)* | *2* | *0* | *0,00* | *0* | *0,0* | *-* |
| *4a* | *(1,1,1)* | *3* | *0* | *0,00* | *0* | *0,0* | *-* |

Table 2. Effects of Comprehensive Management as reflected by the % changes in the different intervention paths between the BCM and ACM cohorts, for each of the four Robson classes considered (1,2a,3,4a). A “-“ sign indicates the cases where the related rate of change was not computable due to lack of data.

| ***Robson class*** | ***Intervention path (x,y,z)*** | ***Number of interventions (x+y+z)*** | ***BCM*** | | ***ACM*** | | ***% change*** |
| --- | --- | --- | --- | --- | --- | --- | --- |
|  |  |  | *Number of women N(x,y,z)* | *% of women f(x,y,z)* | *Number of women N(x,y,z)* | *% of women f(x,y,z)* |  |
| 1 & 3 | *(0,0,0)* | *0* | *347* | *72,75* | *865* | *78,1* | *7,41* |
| 1 & 3 | *(0,0,1)* | *1* | *14* | *2,94* | *32* | *2,9* | *-1,51* |
| 1 & 3 | *(0,1,0)* | *1* | *7* | *1,47* | *32* | *2,9* | *96,98* |
| 1 & 3 | *(0,1,1)* | *2* | *0* | *0,00* | *1* | *0,1* | *-* |
| 1 & 3 | *(1,0,0)* | *1* | *90* | *18,87* | *148* | *13,4* | *-29,14* |
| 1 & 3 | *(1,0,1)* | *2* | *8* | *1,68* | *12* | *1,1* | *-35,37* |
| 1 & 3 | *(1,1,0)* | *2* | *10* | *2,10* | *17* | *1,5* | *-26,75* |
| 1 & 3 | *(1,1,1)* | *3* | *1* | *0,21* | *0* | *0,0* | *-100,00* |
| 2a & 4a | *(0,0,0)* | *0* | *41* | *25,31* | *104* | *38,8* | *53,33* |
| 2a & 4a | *(0,0,1)* | *1* | *11* | *6,79* | *19* | *7,1* | *4,41* |
| 2a & 4a | *(0,1,0)* | *1* | *2* | *1,23* | *4* | *1,5* | *20,90* |
| 2a & 4a | *(0,1,1)* | *2* | *1* | *0,62* | *1* | *0,4* | *-39,55* |
| 2a & 4a | *(1,0,0)* | *1* | *71* | *43,83* | *97* | *36,2* | *-17,42* |
| 2a & 4a | *(1,0,1)* | *2* | *30* | *18,52* | *32* | *11,9* | *-35,52* |
| 2a & 4a | *(1,1,0)* | *2* | *6* | *3,70* | *10* | *3,7* | *0,75* |
| 2a & 4a | *(1,1,1)* | *3* | *0* | *0,00* | *1* | *0,4* | *-* |

Table 3. Effects of Comprehensive Management as reflected by the % changes in the Treatment ratios between the BCM and ACM cohorts, for aggregated classes (1,3) (“spontaneous labour”) and (2a,4a) (“induced labour”) considered together.

**A.2. Simple measures of intensity of medical interventions during labour. Their forms, decomposition and comparisons.**

*Treatment ratios and their representations: OTR and ILI*

As stated in the main text, the most basic measure of the intensity of intervention in a group (e.g., a single Robson class), or cohort, of N women who entered labour, is given by the overall treatment ratio (OTR), here also denoted by *H*. The OTR is computed by dividing the total number (E) of medical treatments of any type experienced by these women, by the number of women in the cohort (N):

$H=\frac{E}{N}$ (1)

By definition H ranges between 0 and the maximum number of possible interventions that a woman can undertake, *n.* By dividing the OTR by *n* one gets the normalized measure termed ILI in the main text:

$ILI=\frac{H}{n}$ (2)

Both the OTR and the ILI are *crude* measures: they aggregate all the treatment events regardless e.g., of their nature and of the characteristics of the exposed women.

*Form 1 and related comparisons*.

Letting N_T_ <N to denote the number of treated women (i.e., women who experienced at least one treatment). Form 1 reported in the main text is obtained by writing:

$H=\frac{N_{T}}{N}\cdot\frac{E}{N_{T}}=S\cdot H_{T}$ (3)

where S denotes the treated proportion and$H_{T}=E/N_{T}$the treatment ratio of treated women.

Suppose now we want to use (3) to compare two different OTRs $H_{1}$and $H_{2}$i.e., to determine which factor between *S* and *H_T_* contributed more to the observed difference: $H_{2}-H_{1}$. Let $S_{2}=S_{1}\left(1+r_{S}\right)$, $H_{T,2}=H_{T,1}\cdot\left(1+r_{H}\right)$, where $r_{S}$ and $r_{HT}$ represent the relative rate of change of *S* and of $H_{T}$respectively. Then, the relative rate of change of the OTR, also termed the *relative difference*, can be expressed as:

$r_{H}=\frac{H_{2}-H_{1}}{H_{1}}=\left(1+r_{S}\right)\left(1+r_{H_{T}}\right)-1=r_{S}+r_{H_{T}}+r_{S}r_{H_{T}}$ (4)

Formula (4) states that the relative rate of change of the OTR can be expanded as the sum of the relative rates of change of *S* and of $H_{T}$, plus the product $r_{S}r_{H_{T}}$ which represents the so called *interaction term.* The latter term arises every time both factors of the OTR are varied. Note that the contribution by the interaction term is positive if the rates of change $r_{S}$ and $r_{HT}$ have the same sign and negative in the opposite case.

*Form 2: type-specific treatment ratios*

Since the total number of treatment events *E* is the sum of number of treatments of the various types: $E=\sum_{j=1}^{n} E_{j}$, it holds

$H=\frac{\sum_{j=1}^{n} E_{j}}{N}=\sum_{j=1}^{n} H_{j}$, (5)

where each $H_{j}$ represents a type-specific treatment ratio (STRs). The simplified classification of interventions reported in the main text (augmentation, ventouse and caesarean section) yields to the simple formula reported therein as Form 2: $H=H_{A}+H_{V}+H_{C}$.

*Form 3: OTR as the average of the distribution of the number of treatments received*.

The distribution of the number of treatments per woman is the discrete distribution J taking on the integer values j=0,1,2,.., n, with absolute (relative) frequencies $N_{j}$ ($f_{j}$) representing the number (proportion) of women who experienced exactly j treatments. Then Form of the main text arises:

$H=\frac{E}{N}=\frac{\sum_{j=0}^{n} j\cdot N_{j}}{N}=\frac{\sum_{j=1}^{n} j\cdot N_{j}}{N}=\sum_{j=1}^{n} j\cdot f_{j}$ (6)

*Form 4:* *OTR as the average of the distribution of number of treatments along the different intervention paths*.

In presence of *n* sequential interventions (as in Figure 1a of main text), the realized intervention path, or history, of each woman is represented by a sequence of length n of binary realizations $\left(x_{1},x_{2},...,x_{n}\right)$, where $x_{i}=0 , i=1,2,..,n$ if that woman did not experience treatment i, and $x_{i}=1$ if the woman experienced treatment i. A woman who followed history $\left(x_{1},x_{2},...,x_{n}\right)$ received $\left(x_{1}+x_{2}+...+x_{n}\right)$ treatments in total. Letting $N\left(x_{1},x_{2},...,x_{n}\right)$ represent the number of women who followed that particular history, and $f\left(x_{1},x_{2},...,x_{n}\right)$ the corresponding relative frequency, one has:

$$E=\sum_{x_{1}=0}^{1} \sum_{x_{2}=0}^{1} ...\sum_{x_{n}=0}^{1} \left(x_{1}+x_{2}+...+x_{n}\right)N\left(x_{1},x_{2},...,x_{n}\right)$$

so that

$H=\frac{E}{N}=\sum_{x_{1}=0}^{1} \sum_{x_{2}=0}^{1} ...\sum_{x_{n}=0}^{1} \left(x_{1}+x_{2}+...+x_{n}\right)\cdot f\left(x_{1},x_{2},...,x_{n}\right)$ (7)

which is Form 4 reported in the main text.

From formula (7) one obtains Form 3 by aggregating histories yielding to the same number of treatments i.e., by considering the convolution $j=x_{1}+x_{2}+...+x_{n}$, where:

$freq\left\{J=j\right\} =\sum_{x_{1}+x_{2}+...+x_{n}=j} ...\sum f\left(x_{1},x_{2},...,x_{n}\right)$ (8)

Moreover, by decomposing along the internal sum in (6) one obtains Form 2

$$\begin{matrix} H=\frac{E}{N}=\sum_{x_{1}=0}^{1} \sum_{x_{2}=0}^{1} ...\sum_{x_{n}=0}^{1} \left(x_{1}+x_{2}+...+x_{n}\right)\cdot f\left(x_{1},x_{2},...,x_{n}\right)= \\ =\sum_{x_{1}=0}^{1} \sum_{x_{2}=0}^{1} ...\sum_{x_{n}=0}^{1} x_{1}\cdot f\left(x_{1},x_{2},...,x_{n}\right)+\sum_{x_{1}=0}^{1} \sum_{x_{2}=0}^{1} ...\sum_{x_{n}=0}^{1} x_{2}\cdot f\left(x_{1},x_{2},...,x_{n}\right)+...+\sum_{x_{1}=0}^{1} \sum_{x_{2}=0}^{1} ...\sum_{x_{n}=0}^{1} x_{n}\cdot f\left(x_{1},x_{2},...,x_{n}\right)= \\ =\sum_{x_{1}=0}^{1} x_{1} \cdot f\left(x_{1}\right)+\sum_{x_{2}=0}^{1} x_{2} \cdot f\left(x_{2}\right)+...+\sum_{x_{n}=0}^{1} x_{n} \cdot f\left(x_{n}\right)= \\ =\sum_{j=1}^{n} H_{j} \end{matrix}$$

*Form 5: decomposing OTRs through progression proportions*

By progression proportions we mean the proportions of women that, during labour, progress from a given treatment to the next one. Let us consider first the case of a fully sequential treatment history where the n possible treatments are ranked in terms of invasiveness (i.e., treatment one is the less invasive, treatment two is the next less invasive etc) and are administered in a strong sequential protocol. An intervention protocol is *strongly sequential* if treatment (j+1) is administered only if all the previous j treatments were. For example, in the case of three interventions considered in the main text (augmentation, ventouse, cesarean section) strong sequentiality would imply that only treatment histories OA, OAV, OAVC are admissible.

Let $Q_{1}>Q_{2}>...>Q_{n}$.. etc, represent the number of women who experienced respectively at least the first treatment, at least the first two treatments etc, out of the cohort of $Q_{0}=N$ women who entered labour. Note that differences between consecutive Q terms define the numbers $N_{i}$ of woman who experience exactly 0,1,2, … treatments (see Form 3): $Q_{0}-Q_{1}=N_{0}, Q_{1}-Q_{2}=N_{1},> Q_{2}-Q_{3}=N_{2}$.

Then the total number of administered treatments can be expressed also as $E=\sum_{j=1}^{n} E_{j}=\sum_{j=1}^{n} Q_{j}$. The OVR can then be represented as follows:

(9)

Previous formula tells that the average number of treatments can be represented as the product of the proportion $p_{01}$ of women who progressed from onset of labour (O) to the first treatment, receiving each one exactly one treatment - this is the meaning of the unit term in the parenthesis - plus the average number ($\tilde{H}_{1}$) of further treatments among these women (as such this is a special case of Form 2). This formula can be further developed by expanding $\tilde{H}_{1}$ as follows

(10)

so that

(11)

Where $p_{12}$ is the proportion of women who progressed to the second treatment among those received the first one and $\tilde{H}_{2}$ is the average number of further treatments i.e., beyond the second, among women who received the first two treatments.

By iterating we end up with the general formula:

$H=p_{01}\cdot\left(1+p_{12}\cdot\left(1+p_{13}\cdot....\left(1+p_{m-1,m}\right)\right)\right)$ (12)

The latter formula evidences that under a strong sequential intervention protocol the OVR can be expressed sequentially by the sum of the proportions progressing to the next treatment from current one counting exactly one treatment at each new step.

Formula (12) can ultimately be expanded as:

$H=p_{01}+p_{01}\cdot p_{12}+p_{01}\cdot p_{12}\cdot p_{13}+...+p_{01}\cdot p_{12}\cdot...\cdot p_{m-1,m}$ (13)

or, using a slightly less involved notation just acknowledging the existence of n distinct progression proportions:

$H=p_{1}+p_{1}\cdot p_{2}+p_{1}\cdot p_{2}\cdot p_{3}+...+p_{1}\cdot p_{2}\cdot...\cdot p_{n}=\sum_{i=1}^{n} \prod_{j=1}^{i} p_{j}$ (14)

Formula (14), which is commonly used e.g., in demography to represent the average number of children per woman in a cohort (the so called cohort *total fertility rate*) by emphasizing the woman’s parity, shows that in a strong sequential treatment history the OTR is entirely determined by the sole (conditional) proportions to progress to the next treatment having experienced the current one. These quantities represent natural estimates for the underlying *progression probabilities*. The *strong sequential* case is a special one which restricts the number of possible treatment histories: it implies indeed that women who entered treatment j, necessarily experienced before all previous treatments (*1,2,..,j-1*), that is to say their history was “obligate”.

Extension to *weakly* sequential treatment protocols, as those considered in the main text (augmentation, ventouse and caesarean) is reported below. A simple way to understand the point is by using the type-specific treatment ratios written as follows:

$\begin{matrix} H_{A}=p_{OA} \\ H_{V}=p_{OV}+p_{OAV} \\ H_{C}=p_{OC}+p_{OAC}+p_{OVC}+p_{OAVC} \end{matrix}$ (15)

The previous relations are intuitive. For example, the TR for ventouse ($H_{V}$) is given by the sum of the proportion $p_{OV}$ of women who entered labour and progressed directly to ventouse (receiving exactly one treatment), plus the proportion $p_{OAV}$ of women who also experienced augmentation before the ventose. Similarly, the TR for CS is the sum of the proportion $p_{OC}$ of women who entered labour and progressed directly to CS (received exactly one treatment), plus eventually the proportion $p_{OAVC}$ of women who ended in CS after having experienced both augmentation and ventose.

Noting that these proportions represent straightforward estimates of the underlying probabilities that the generic woman follows the treatment history considered.

By using the product rule of probability one can write previous formulas as products of conditional probabilities e.g.,

$$p_{OAVC}=P\left(A\right)P\left(V|A\right)P\left(C|A,V\right)$$

This expression reminds that in the general case a “dependency on history” will appear. A sharp simplification occurs by resorting to the Markov hypothesis, which amounts to assume that the probability to move from a given intervention to the next one only depends on being in the present intervention and not on the prior treatment history. Under this hypothesis, for example the probability $p_{OAVC}$ of experiencing all available treatments (A,V,C) can be factored as the product $p_{OA}p_{AV}p_{VC}$ of the probabilities of experiencing augmentation first, times the conditional probability of progressing to ventose after augmentation, times the conditional probability of progressing to cesarean section after ventose. The following relations hold:

$$H_{A}=p_{OA}$$

$$H_{V}=p_{OV}+p_{OA}p_{AV}$$

$H_{C}=p_{OC}+p_{OA}p_{AC}+p_{OV}p_{VC}+p_{OA}p_{AV}p_{VC}$ (16)

Form 5 for the OTR reported in Box 1 of the main text is then obtained by taking the sum $H=H_{A}+H_{V}+H_{C}$.

The general weakly sequential case requires to consider many more transitions. Even assuming markovianity this requires a more cumbersome notation:

$H=\sum_{i=1}^{n} p_{0i}+\sum_{i=1}^{n-1} \sum_{j=i}^{n} p_{0i}p_{ij}+\sum_{i=1}^{n-2} \sum_{j=i}^{n-1} \sum_{l=j}^{n} p_{0i}p_{ij}p_{jl}+etc$ (17)

**2. Assessing the contributions of the different progression proportions to the rate of change of a treatment ratio: an alternative approach**

In the main text, we have assessed the relative contributions of the different PPs to the difference $H_{2}-H_{1}$between two OTRs by using the simple stepwise replacement algorithm. Here we rely on the simplified case of strong sequential treatment protocols to propose, as an alternative, a general decomposition of the relative difference $r_{H}=\left(H_{2}-H_{1}\right)/H_{1}$ for OTRs written in the form (14). The issue of comparing such forms for fully sequential histories has been considered in demographic analysis (e.g., Pullum et al 1989, Canudas Romo 2003), who used the total prime differential for identifying the parity progression ratios that most contributed to the difference between two total fertility rates. The approach used here includes the one used by Pullum 1989 as a special case.

As a departure point let us write the OTR as a generic multi-variable function of the different progression proportions:

$H=f\left(p_{1},p_{2},...p_{m}\right)=p_{1}+p_{1}\cdot p_{2}+p_{1}\cdot p_{2}\cdot p_{3}+...+p_{1}\cdot p_{2}\cdot...\cdot p_{m}$ (2.1)

Let us compare two generic OTRs $H_{1},H_{2}$ whose progression proportions are denoted, for sake of notational simplicity, as $x_{1},x_{2},...\cdot,x_{m}$ and $y_{1},y_{2},...\cdot,y_{m}$ i.e.:

$\begin{matrix} H_{1}=x_{1}+x_{1}\cdot x_{2}+...+x_{1}\cdot x_{2}\cdot...\cdot x_{m} \\ H_{2}=y_{1}+y_{1}\cdot y_{2}+...+y_{1}\cdot y_{2}\cdot...\cdot y_{m} \end{matrix}$ (2.2)

Let us moreover denote$y_{i}=x_{i}\left(1+r_{i}\right)$ where $r_{i}$ represents the relative rate of change along the i-th dimension. It is easy to show that the relative rate of change in the treatment ratio $r_{H}$ can be written in terms of the relative rates of change $r_{i}$ and their interactions of the various orders, with specific weighting quantities. Indeed, it holds $r_{H}=\left(H_{2}/H_{1}\right)-1$ where:

$$\begin{matrix} \frac{H_{2}}{H_{1}}=\frac{y_{1}+y_{1}\cdot y_{2}+y_{1}\cdot y_{2}\cdot y_{3}+....}{x_{1}+x_{1}\cdot x_{2}+x_{1}\cdot x_{2}\cdot x_{3}+....}=\frac{y_{1}}{x_{1}}\cdot\frac{x_{1}}{H_{1}}+\frac{y_{1}\cdot y_{2}}{x_{1}\cdot x_{2}}\cdot\frac{x_{1}\cdot x_{2}}{H_{1}}+\frac{y_{1}\cdot y_{2}\cdot y_{3}}{x_{1}\cdot x_{2}\cdot x_{3}}\cdot\frac{x_{1}\cdot x_{2}\cdot x_{3}}{H_{1}}+...= \\ =\frac{y_{1}}{x_{1}}\cdot w_{x,1}+\frac{y_{1}\cdot y_{2}}{x_{1}\cdot x_{2}}\cdot w_{x,2}+\frac{y_{1}\cdot y_{2}\cdot y_{3}}{x_{1}\cdot x_{2}\cdot x_{3}}\cdot w_{x,3}+... \end{matrix}$$

involving the quantities:

$$w_{x,h}=\frac{x_{1}\cdot x_{2}\cdot...\cdot x_{h}}{H_{1}}$$

which are normalized weights because they are non-negative with:

$\sum_{h=1}^{n} w_{x,h}=\frac{H_{1}}{H_{1}}=1$.

Letting $y_{i}=x_{i}\left(1+r_{i}\right)$ we get:

$$\frac{H_{2}}{H_{1}}=\left(1+r_{1}\right)\cdot w_{x,1}+\left(1+r_{1}\right)\left(1+r_{2}\right)\cdot w_{x,2}+...+\left(1+r_{1}\right)\left(1+r_{2}\right)...\left(1+r_{n}\right)\cdot w_{x,n}$$

By re-arranging terms:

$$\begin{matrix} \frac{H_{2}}{H_{1}}=\sum_{h=1}^{n} w_{x,h}+r_{1}\sum_{h=1}^{n} w_{x,h}+r_{2}\sum_{h=2}^{n} w_{x,h}+...+r_{n}\sum_{h=n}^{n} w_{x,h}+ \\ +r_{1}r_{2}\sum_{h=2}^{n} w_{x,h}+r_{1}r_{3}\sum_{h=3}^{n} w_{x,h}+....+r_{n-1}r_{n}\sum_{h=n}^{n} w_{x,h}+...+r_{1}r_{2}...r_{n}\sum_{h=n}^{n} w_{x,h} \end{matrix}$$

Thus we eventually obtain:

$\begin{matrix} r_{H}=\frac{H_{2}}{H_{1}}=\sum_{h=1}^{n} w_{x,h}+r_{1}\sum_{h=1}^{n} w_{x,h}+r_{2}\sum_{h=2}^{n} w_{x,h}+...+r_{n}\sum_{h=n}^{n} w_{x,h}+ \\ +r_{1}r_{2}\sum_{h=2}^{n} w_{x,h}+r_{1}r_{3}\sum_{h=3}^{n} w_{x,h}+....+r_{n-1}r_{n}\sum_{h=n}^{n} w_{x,h}+...+r_{1}r_{2}...r_{n}\sum_{h=n}^{n} w_{x,h} \end{matrix}$ (2.4)

From (2.4) one can evaluate the contributions of the various dimensions to the overall change by considering first the contributions by the rate of change along the single (“marginal”) dimensions, then by considering the contribution of pairwise marginal dimensions i.e., by pairs of dimensions, etc. In particular, when we look at the contributions of the single marginal dimensions, we get:

$r^{H}=\sum_{t=1}^{n} r_{t}\left(\sum_{j=t}^{n} w_{x,j}\right)$ (2.5)

where it holds:

$$W_{t}=\sum_{j=t}^{m} w_{x,j}>W_{t+1}$$

Equation (14) keeps an intuitive feeling, namely that the overall rate of change of the treatment ratio is the sum of the relative rates of change over the various dimensions “weighted” by the declining non-normalized weights $W_{t}$.

This formulation leads to a very simple criterion for assessing the contributions from the different progression proportions, by simply taking the proportions to the overall relative change of H that are determined by each single dimension (that is, when the other dimensions are set to zero). For example, the contribution by $r_{1}$ would be given by:

$c_{1}^{H}=\frac{r_{1}\cdot\left(w_{x,1}+w_{x,2}+w_{x,3}\right)}{r_{1}\cdot\left(w_{x,1}+w_{x,2}+w_{x,3}\right)+r_{2}\cdot\left(w_{x,2}+w_{x,3}\right)+r_{3}\cdot w_{x,3}}$ (2.6)

In particular, the previous formulation quickly gives the main property that reductions in earlier treatments are always more effective – coeteris paribus - in containing the overall intensity than reductions in later treatments. The simplest way to show this is by considering the case of identical relative reductions over each dimension i.e. $r_{1}=r_{2}=r_{3}=...=r$, in which case from (2.6) it follows simply:

$$c_{i}^{H}=\frac{r_{i}W_{i}}{\sum_{t=1}^{m} r_{t}W_{t}}=\frac{W_{i}}{\sum_{t=1}^{m} W_{t}}>c_{i+1}^{H}$$

**References for Appendix**

Andreev EM, Shkolnikov VM, Begun AZ (2002) Algorithm for decomposition of differences between aggregate demographic measures and its application to life expectancies, healthy life expectancies, parity-progression ratios and total fertility rates. Demographic Research – Volume 7, Article 14, http://www.demographic-research.org 499

Preston SH, Heuveline P, Guillot M. Demography: Measuring and Modeling Population Processes.

Blackwell Publishing. London. 2001.

Canudas Romo V. (2003) Decomposition Methods in Demography, PhD Thesis, University of Groningen.

Pullum TW, Tedrow LM, Herting JR (1989), Measuring Change and Continuity in Parity Distributions, Demography, Vol. 26, No. 3, 485-498.
